# Omadacycline for the treatment of patients with *Legionella pneumophila* pneumonia after experiencing liver dysfunction: case series

**DOI:** 10.3389/fmicb.2024.1408443

**Published:** 2024-06-12

**Authors:** Ani Zhu, Qian Ma, Zhiyan Liu

**Affiliations:** Department of Pulmonary and Critical Care Medicine, Xi’an No. 3 Hospital, The Affiliated Hospital of Northwest University, Xi’an, China

**Keywords:** *Legionella pneumophila*, pneumonia, omadacycline, liver function, case series

## Abstract

**Introduction:**

Antibiotics frequently induce abnormal liver function. Omadacycline is a novel aminomethylcycline antibiotic, which shows potent activity against Gram-positive and Gram-negative aerobic, anaerobic, and atypical (including *Legionella pneumophila*) bacteria. Of note, omadacycline is tolerable in most patients with liver impairment. However, evidence regarding the application of omadacycline in patients with *Legionella pneumophila* pneumonia after experiencing liver dysfunction is scarce.

**Methods:**

The current study reported 6 cases of patients with *Legionella pneumophila* pneumonia receiving omadacycline as subsequent antibiotics after experiencing liver dysfunction.

**Results:**

These 6 cases were admitted to the hospital for pneumonia and received antibiotic therapy, including piperacillin-tazobactam, imipenem, meropenem, and moxifloxacin. After receiving these antibiotics, increased liver enzymes were noted. Although hepatoprotective therapy (such as magnesium isoglycyrrhizinate and glutathione) was given, the liver function was still abnormal. According to metagenomic next-generation sequencing, these patients were diagnosed with *Legionella pneumophila* pneumonia. Considering the abnormal liver function, the antibiotic therapy was switched to omadacycline-containing antibiotic therapy. After that, liver function was improved, and the infection was ameliorated. Ultimately, all patients discharged from the hospital, including 2 patients who achieved complete clinical symptomatic improvement and 4 patients who achieved partial clinical symptomatic improvement.

**Discussion:**

This study emphasizes the successful treatment of switching to omadacycline after experiencing abnormal liver function in patients with *Legionella pneumophila* pneumonia. This study suggests that omadacycline may serve as an optional antibiotic for patients with *Legionella pneumophila* pneumonia, especially when occurring liver dysfunction. However, more clinical studies are required to validate our findings.

## 1 Introduction

*Legionella pneumophila* is a major species of *Legionellaceae*, which causes severe Legionnaires’ disease characterized by pneumonia (Iliadi et al., 2022). *Legionella pneumophila* infection accounts for 2–9% of cases of community-acquired pneumonia (Cunha et al., 2016). It is recommended to test *Legionella pneumophila* at hospital admission for patients with community-acquired or hospital-acquired pneumonia (Phin et al., 2014; Allgaier et al., 2021; Gattuso et al., 2022). Generally, the first-line antibiotics for patients with *Legionella pneumophila* pneumonia include fluoroquinolone (such as levofloxacin or moxifloxacin) and macrolide (such as azithromycin) (Viasus et al., 2022). However, consistent use of these antibiotic agents may induce abnormal liver function in patients with *Legionella pneumophila* pneumonia (Azithromycin, 2012; Mohsen et al., 2020; Rezahosseini et al., 2022). Therefore, optional antibiotics with less effects on liver function should be further investigated in patients with *Legionella pneumophila* pneumonia.

Omadacycline is a novel aminomethylcycline antibiotic, which possesses broad-spectrum antibacterial activity against Gram-positive and Gram-negative aerobic, anaerobic, and atypical bacteria (The Medical Letter, 2021; Leviton and Amodio-Groton, 2022; Davis et al., 2023; Sakoulas et al., 2023). Regarding *Legionella pneumophila* infection, a previous study reports that omadacycline shows comparable efficacy to moxifloxacin in patients with *Legionella pneumophila* infection (Surani et al., 2020). Apart from that, omadacycline is tolerable for most patients with liver function impairment (Opal et al., 2019; Kovacs et al., 2020). Hence, it is hypothesized that omadacycline may serve as an optional antibiotic for patients with *Legionella pneumophila* pneumonia after experiencing abnormal liver function. However, relevant evidence is scarce. The current study reported 6 cases of patients with *Legionella pneumophila* pneumonia who switched to omadacycline after experiencing abnormal liver function, with the aim of providing evidence for the clinical use of omadacycline.

## 2 Case presentation

### 2.1 Case 1

A 59-year-old male was admitted to the hospital on September 14th, 2022, due to a fever for 2 days. This patient had a fever with a temperature of 39°C. Computed tomography (CT) showed inflammation in the lower lobe of the right lung, tiny nodules in the upper lobes of both lungs, fibrous cords in the upper lobe of the left lung and in the lower lobe of the right lung, limited emphysema in the upper lobe of the right lung, and large pulmonary alveoli. Meanwhile, the COVID-19 testing was negative. The samples of this patient were taken for sputum smear, sputum culture, and respiratory tract antibody testing in the blood. Since the *Legionella pneumophila* urine antigen test was unavailable in our hospital, this patient did not undergo the urine antigen test. This patient was initially diagnosed with pneumonia of the lower lobe of the right lung. The initial antibiotic therapy was piperacillin-tazobactam. On September 15th, the patient was found to have an intermittent high fever (highest temperature: 39.5°C) and an increased area of lung infection, and mycoplasma infections. The suspicion of mycoplasma infections was based on the following consideration: after admission, the patient still had a fever despite treatment with piperacillin-tazobactam. Laboratory test results indicated a positive mycoplasma pneumonia antibody. Additionally, considering that mycoplasma pneumonia was a common pathogen in the etiological spectrum of community-acquired pneumonia in China, there was a suspicion of the presence of mycoplasma infections. The antibiotic therapy was adjusted to piperacillin-tazobactam combined with moxifloxacin. Meanwhile, the patient was found to have abnormal liver function [alanine aminotransferase (ALT): 63 U/L; aspartate aminotransferase (AST): 52 U/L], which might be due to infection and fatty liver. Therefore, glutathione combined with magnesium isoglycyrrhizinate was given for hepatoprotective therapy. On September 16th, the patient had an intermittent fever yesterday (the highest temperature: 39°C). In addition, the patient was treated with bronchoscopy and alveolar lavage, and alveolar lavage fluid was collected for metagenomic next-generation sequencing (mNGS) examination. On September 18th, elevated liver enzymes [ALT: 162 U/L; AST: 145 U/L; γ-glutamyl transferase (GGT): 233 U/L] were noted, and drug-induced liver dysfunction was suspected. On September 19th, the patient’s temperature had returned to normal for the last 3 days. The mNGS suggested *Legionella pneumophila* infection. Considering the abnormal liver function, the antibiotic therapy was adjusted to omadacycline plus piperacillin-tazobactam, and hepatoprotective therapy was maintained. Even 6 days after switching to omadacycline patient remained afebrile, and his clinical symptoms and liver function tests (ALT: 60 U/L; GGT: 146 U/L) were improved. On September 26th, the patient was discharged. This patient was instructed to continue omadacycline at 300 mg once daily for 7 days after discharge.

### 2.2 Case 2

A 79-year-old female was admitted to the hospital on August 23rd, 2022, due to an intermittent cough with vague pain in the left lower quadrant of the chest for 10 days. CT (examined on August 17th) showed inflammation in the lower lobe of the left lung and a small amount of pleural effusion on the left side, interstitial changes with scattered fibrous streak foci in both lungs, and calcified foci in the upper lobe of the left lung. On August 22nd, CT suggested a small amount of fluid in the left pleural cavity, a more severe left lung lesion than before (August 17th), and a new lesion found on the right side. Meanwhile, the COVID-19 testing was negative. The samples of this patient were taken for sputum smear, sputum culture, and respiratory tract antibody testing in the blood. Since the *Legionella pneumophila* urine antigen test was unavailable in our hospital, this patient did not undergo the urine antigen test. This patient was diagnosed with double pneumonia. The initial antibiotic therapy was imipenem combined with moxifloxacin. On August 24th, the patient’s temperature was normal, and clinical symptoms were ameliorated, but liver dysfunction was noted [ALT: 74 U/L; AST: 81 U/L; total protein (TP): 52.2 g/L; albumin (ALB): 21.3 g/L]. Therefore, initial antibiotic therapy was maintained, and glutathione was added for liver protection. On August 25th, the temperature was normal. Meanwhile, this patient was treated with bronchoscopy and alveolar lavage, and alveolar lavage fluid was collected for mNGS examination. On August 26th, the temperature remained normal. The mNGS suggested *Legionella pneumophila* and *Candida glabrata* infections. Therefore, fluconazole gargle was given, moxifloxacin was continued, and imipenem was discontinued. Considering the severity of the patient’s lung infection and the uncertainty about colonizing and infecting bacteria, fluconazole was administered for gargling to prevent oral fungal infection. Intravenous fluconazole was not administered. After subsequent treatments, the patient’s symptoms were improved, suggesting that *Candida glabrata* was likely a contaminant or oral colonizing bacteria. On August 27th, the temperature was normal. However, this patient’s liver function (ALT: 104 U/L; AST: 129 U/L; TP: 59.1 g/L; ALB: 26.1 g/L) was still abnormal. Hence, moxifloxacin was discontinued, and omadacycline was administrated. On September 5th, the liver function was improved (TP: 63.5 g/L; ALB: 35.7 g/L), and the patient’s clinical symptoms were found to be obviously better. However, there was a temporary shortage of omadacycline in our hospital. Considering the patient’s liver function was enhanced, after communicating with the family members, moxifloxacin was prescribed for this patient. Meanwhile, omeprazole was administered to protect the liver function, and the liver function was closely monitored. On September 8th, the clinical symptoms of this patient were basically ameliorated, and she was discharged on this day. Considering the possible recurrence of liver function impairment due to subsequent moxifloxacin, this patient was instructed to switch to omadacycline after discharge. Additionally, after communicating with the patient’s family members, they requested a medication with fewer side effects on the liver, and omadacycline was available in the local pharmacy. Therefore, the patient was instructed to continue omadacycline at 300 mg once daily for 7 days after discharge.

### 2.3 Case 3

A 45-year-old male was admitted to the hospital on August 4th, 2022, due to a cough for 5 days and fever for 1 day. The temperature of this patient was 35.8°C on this day. CT showed large solid exudative shadows in the right lung, especially in the right lower lung, a little pleural effusion on the right side, and enlarged mediastinal lymph nodes. The COVID-19 testing was negative. The samples of this patient were taken for sputum smear, sputum culture, and respiratory tract antibody testing in the blood. Since the *Legionella pneumophila* urine antigen test was unavailable in our hospital, this patient did not undergo the urine antigen test. This patient was diagnosed with right lung pneumonia. The initial antibiotic therapy was meropenem. On August 5th, the patient’s temperature was normal, but abnormal liver function was noted (ALT: 90 U/L; AST: 378 U/L; GGT: 272 U/L; ALB: 32.4 g/L). Therefore, antibiotic therapy with meropenem was continued, and magnesium isoglycyrrhizinate was added for liver protection. On August 9th, the patient had a fever, and the highest temperature was 38.2°C on August 8th. Liver function indexes remained abnormal (ALT: 105 U/L; AST: 193 U/L; GGT: 263 U/L; ALB: 27.6 g/L). Meropenem was maintained as antibiotic therapy, and glutathione was further added for hepatoprotective therapy. Meanwhile, this patient was treated with bronchoscopy and alveolar lavage, and alveolar lavage fluid was collected for mNGS examination. On August 10th, the patient’s temperature returned to normal. The mNGS suggested *Legionella pneumophila* infection. Meanwhile, considering the situation of liver dysfunction, meropenem was discontinued, and antibiotic therapy was adjusted to omadacycline. On August 16th, the temperature was normal, clinical symptoms were completely ameliorated, and the liver enzymes were reduced (ALT: 77 U/L; AST: 60 U/L; GGT: 141 U/L), omadacycline and hepatoprotective therapy were maintained. The patient was discharged on August 17th. The patient was instructed to continue omadacycline at 300 mg once daily for 7 days after discharge.

### 2.4 Case 4

A 33-year-old male was admitted to the hospital on August 8th, 2022, due to a fever for 3 days and a cough for 1 day. Of note, this patient had a fever with a maximum temperature of 39.8°C after choking from swimming 3 days ago. The temperature of this patient was 39.7°C on this day. CT showed scattered patchy hyperdense shadows in both lungs with blurred edges and localized solid changes in lung tissue, prominent on the right side, and fatty liver. The COVID-19 testing was negative. The samples of this patient were taken for sputum smear, sputum culture, and respiratory tract antibody testing in the blood. Since the *Legionella pneumophila* urine antigen test was unavailable in our hospital, this patient did not undergo the urine antigen test. He was diagnosed with double pneumonia. The initial antibiotic therapy was meropenem. On August 9th, this patient still had a fever with the highest temperature of 39°C. In addition, atypical mycoplasma infections were considered because the patient presented with a cough and a fever after choking from swimming. Therefore, antibiotic therapy with moxifloxacin was empirically added. Meanwhile, the liver function was abnormal [prothrombin time (PT): 8.5 s; PT activity: 151], and glutathione was given for liver protection. On August 10th, fever still existed (highest temperature: 39.2°C), and elevated liver enzymes were noted (ALT: 93 U/L; AST: 43 U/L; GGT: 204 U/L). Then, moxifloxacin was discontinued, and magnesium isoglycyrrhizinate was further added for liver protection. On the same day, this patient was treated with bronchoscopy and alveolar lavage, and alveolar lavage fluid was collected for mNGS examination. On August 13th, the patient’s temperature returned to normal. The mNGS suggested *Legionella pneumophila, Corynebacterium striatum, Tropheryma whipplei, Mycobacterium tuberculosis complex,* and *Hemophilus influenza* infections. *Haemophilus influenza* pneumonia manifested as multifocal lobular pneumonia in both lungs, which tended to affect the periphery. The chest CT findings of *Haemophilus influenza* pneumonia typically included ground-glass opacity, centrilobular nodules, consolidation, and bronchial wall thickening, while lobular consolidation was less common. In this case, the patient presented with mainly consolidative exudate, and the imaging findings suggested *Legionella pneumophila* as the pathogen. Additionally, as a young male with community-acquired pneumonia and without the presence of drug-resistant bacteria, meropenem should be effective against *Haemophilus influenzae*. However, the response to meropenem was unsatisfactory in this patient, suggesting that *Haemophilus influenza* was not the pathogen. Notably, the mNGS showed *Mycobacterium tuberculosis complex*, but the temperature was normal after the current antibiotic therapy, and the T-spot and purified protein derivative were negative. Therefore, this patient was unlikely to be infected with tuberculosis. Then, it was speculated that *Legionella pneumophila* might be the main causative pathogen. Meanwhile, the liver function was still abnormal (ALT: 228 U/L; AST: 96 U/L; ALB: 35.2 g/L). Hence, omadacycline was added, and meropenem was continued; meanwhile, hepatoprotective therapy was maintained. On August 19th, the temperature was normal, the patient’s liver function was enhanced (ALT: 112 U/L; GGT: 110 U/L), and the clinical symptoms were completely ameliorated. Ultimately, the patient was discharged. The patient was instructed to continue omadacycline at 300 mg once daily for 7 days after discharge.

### 2.5 Case 5

A 65-year-old female was admitted to the hospital on September 3rd, 2022, due to a fever for 3 days. The patient had a fever with a temperature of 38.9°C. CT showed inflammation of the lower lobe of the right lung. The COVID-19 testing was negative. The samples of this patient were taken for sputum smear, sputum culture, and respiratory tract antibody testing in the blood. Since the *Legionella pneumophila* urine antigen test was unavailable in our hospital, this patient did not undergo the urine antigen test. This patient was diagnosed with pneumonia of the lower lobe of the right lung. The initial antibiotic therapy was piperacillin-tazobactam. On September 4th, the highest temperature was 39.6°C, and the patient’s liver enzymes were abnormal (ALT: 86 U/L; AST: 97 U/L). Of note, this patient was receiving oral atorvastatin due to coronary atherosclerosis and aortic sclerosis. Considering abnormal liver function, oral atorvastatin was discontinued, and magnesium isoglycyrrhizinate was given for liver protection. The antibiotic therapy was maintained. On September 5th, this patient still had intermittent fevers (the highest temperature was 39.6°C), and the antibiotic therapy was adjusted to meropenem plus moxifloxacin. Meanwhile, glutathione was further added for liver protection. On September 6th, the patient was treated with bronchoscopy and alveolar lavage, and alveolar lavage fluid was collected for mNGS examination. On September 8th, the patient’s temperature returned to normal. The mNGS suggested *Legionella pneumophila* infection. Regarding liver enzymes, ALT was increased to 116 U/L, AST was decreased to 53 U/L, and GGT was 264 U/L. Thus, meropenem and moxifloxacin were discontinued, and the antibiotic therapy was switched to omadacycline. On September 13th, the temperature was normal, the clinical symptoms of this patient were basically improved, and the liver function (ALT: 71 U/L; AST: 33 U/L; GGT: 185 U/L) was also ameliorated. Hence, the patient was scheduled for discharge. The patient was instructed to continue omadacycline at 300 mg once daily for 7 days after discharge.

### 2.6 Case 6

A 44-year-old male was admitted to the hospital on October 21st, 2022, due to a cough with sputum and fever for 4 days. The temperature of this patient was 36.8°C on this day. CT showed lobar pneumonia of the lower lobe of the right lung. The COVID-19 testing was negative. The samples of this patient were taken for sputum smear, sputum culture, and respiratory tract antibody testing in the blood. Since the *Legionella pneumophila* urine antigen test was unavailable in our hospital, this patient did not undergo the urine antigen test. He was diagnosed with pneumonia of the lower lobe of the right lung. The initial antibiotic therapy was piperacillin-tazobactam. On October 22nd, this patient had a fever with the highest temperature of 39.6°C. Laboratory tests showed that the patient had abnormal liver function [direct bilirubin (DBIL): 8.5 μmol/L; indirect bilirubin (IBIL): 17.4 μmol/L; ALT: 57 U/L; AST: 131 U/L], abnormal renal function [creatinine (Cr): 112 μmol/L], a high myocardial enzyme profile [AST: 123 U/L; creatine kinase (CK): 3842 U/L; creatine kinase isoenzymes (CKMB): 53 U/L; lactate dehydrogenase (LDH): 900 U/L; α-hydroxybutyrate dehydrogenase (HBDH): 637 U/L], and multisystem damage. Therefore, *Legionella pneumophila* infection was considered, and omadacycline was given for antibiotic therapy, along with magnesium glycyrrhizinate for liver protection. The consideration of *Legionella pneumophila* infection was based on Cunha’s Winthrop-University Hospital’s weighted point system (WUH system) and the 2016 Chinese Guidelines for Diagnosis and Treatment of Community-Acquired Pneumonia in Adults. According to WUH system, six predictive factors of *Legionella pneumophila* infection were: (1) temperature > 38.9°C (with relative bradycardia); (2) erythrocyte sedimentation rate > 90 mm/h or C-reactive protein >180 mg/L; (3) ferritin higher than twice the normal level; (4) hypophosphatemia; (5) creatine kinase elevation >2 times the normal level; (6) microscopic hematuria upon admission. If more than 3 of these factors were present, *Legionella pneumophila* infection was highly suspected. In this case, the patient met criteria (1), (2), (4), and (5). According to the 2016 Chinese Guidelines for Diagnosis and Treatment of Community-Acquired Pneumonia in Adults, adult community-acquired pneumonia patients present with relatively bradycardic fever, acute onset of headache, non-drug-induced impaired consciousness or lethargy, non-drug-induced diarrhea, shock, acute liver and kidney dysfunction, hyponatremia, hypophosphatemia, and lack of response to beta-lactam antibiotics, *Legionella pneumophila* infection should be considered. Based on the above considerations, this patient was diagnosed with *Legionella pneumophila* infection before mNGS results. On October 23rd, the patient was found to have abnormal mental status, a high fever, and abnormal liver function (ALT: 59 U/L; AST: 133 U/L; TP: 57.3 g/L; ALB: 33.3 g/L). Although *Legionella pneumophila* infection was considered, co-infections could not be excluded. Then, the antibiotic therapy was adjusted to omadacycline combined with meropenem, and the hepatoprotective therapy was maintained. After the onset of the patient’s mental abnormalities, magnetic resonance imaging (MRI) plus diffusion-weighted imaging (DWI) of the brain was conducted. The results showed that there were small strip-shaped long T1 and long T2 signal shadows in the splenium of the corpus callosum, with high T2 Flair signals, and the DWI showed limited diffusion and high signals. Abnormal signal foci are observed in the splenium of the corpus callosum, suggesting reversible splenial lesion syndrome. *Legionella pneumophila* infection might cause reversible splenial lesion syndrome; the blood cultures were negative after admission, and the patient’s hemodynamics were stable. Therefore, sepsis was not considered. On October 25th, the patient was treated with bronchoscopy and alveolar lavage, and alveolar lavage fluid was collected for mNGS examination. It was found that the liver function was not obviously improved [ALT: 65 U/L; AST: 115 U/L; GGT: 81 U/L; TP: 57.9 g/L; ALB: 32.5 g/L; alkaline phosphatase (ALP): 128 U/L]. In addition, the patient had a persistent high fever (temperature: 39.7°C), and a combined viral infection was considered. Therefore, ganciclovir combined with oseltamivir phosphate was given as antiviral therapy. The patient tested negative for influenza A and B antigens, but positive for cytomegalovirus (CMV) IgG antibodies and negative for CMV IgM antibodies. Given the patient’s repeated high fever and the fact that the infection occurred during a high-risk season for viral infections, a combined viral infection could not be ruled out, and there might be a false-negative result in the virus test. Therefore, the virus-related treatment was empirical. Cerebrospinal fluid examination showed no obvious abnormalities, ruling out intracranial infection. The previous mental status abnormalities might be due to *Legionella pneumophila* infection. At that time, the patient’s mental status returned to normal, and the current antibiotic therapy was maintained. On October 26th, this patient still had an intermittent fever. The mNGS suggested *Legionella pneumophila* infection, and the antibiotic therapy maintained omadacycline only, while meropenem, ganciclovir, and oseltamivir phosphate were discontinued. Glutathione was added to hepatoprotective therapy. On October 28th, the temperature remained normal for 2 days, and the liver function was ameliorated (ALT: 73 U/L; AST: 86 U/L; TP: 54.7 g/L; ALB: 31 g/L). On November 1st, the clinical symptoms of this patient were basically improved, liver function was ameliorated (ALT: 68 U/L; AST: 52 U/L), and the patient was discharged.

## 3 Discussion

*Legionella pneumophila* is a crucial cause of community-acquired pneumonia, which could also affect the heart, brain, abdomen, and joints (Cunha et al., 2016; Iliadi et al., 2022). Omadacycline is a novel antibiotic agent with a wide antibacterial spectrum, while its application for *Legionella pneumophila* infection is only reported in two studies (Surani et al., 2020; Lu et al., 2023). One study indicates that omadacycline is comparable to moxifloxacin for the treatment of *Legionella pneumophila* infection (Surani et al., 2020). Another study presents a case of a patient with *Legionella pneumophila* infection who is successfully treated with omadacycline (Lu et al., 2023). In this study, we reported 6 cases of patients with *Legionella pneumophila* pneumonia. It was found that omadacycline-containing antibiotic therapy successfully ameliorated the clinical symptoms of patients with *Legionella pneumophila* pneumonia. The possible reasons might be that: (1) Omadacycline could inhibit bacterial protein synthesis by interacting with the primary tetracycline binding site on the 30S subunit of the bacterial ribosome, which showed potent activity against atypical pathogens, including *Legionella pneumophila* (Heidrich et al., 2016; Karlowsky et al., 2019; Zhanel et al., 2020). (2) *Legionella pneumophila* was an intracellular pathogen, and omadacycline had a strong ability to penetrate cell membranes, which allowed it to effectively eliminate *Legionella pneumophila* (Dubois et al., 2020). Therefore, these 6 cases were successfully treated by omadacycline-containing antibiotic therapy.

Antibiotics usually induce several adverse events, and abnormal liver function is a common type (Gu et al., 2023). In this study, all 6 patients with *Legionella pneumophila* pneumonia experienced abnormal liver function after receiving initial antibiotics, including piperacillin-tazobactam, imipenem, meropenem, and moxifloxacin. Therefore, close monitoring of liver enzymes is required for patients with pneumonia when initiating antibiotics. Surprisingly, liver function was improved after using omadacycline in patients with *Legionella pneumophila* pneumonia. A potential explanation would be that omadacycline did not undergo hepatic metabolism (Omadacycline, 2012; Rodvold et al., 2020). Thus, omadacycline would not cause hepatotoxicity. Another interesting phenomenon of this study was that abnormal mental status was discovered in case 6. Since cerebrospinal fluid examination showed no abnormalities, intracranial infection was excluded. Therefore, it was suspected that the abnormal mental status might be induced by *Legionella pneumophila* infection. Clinically, physicians should pay more attention to the mental status of patients with *Legionella pneumophila* pneumonia, and corresponding management should be performed to encounter this symptom.

Bronchoscopy and alveolar lavage were performed in 6 patients, and alveolar lavage fluid was collected for mNGS examination. Bronchoscopy is invasive and could be avoided by using classical diagnostic methods, such as sputum examination and serological testing. However, in complex or unclear cases, bronchoscopy and alveolar lavage allow for direct sampling of the lower respiratory tract, which may increase the accuracy of detecting the underlying pathogen [a, b]. It should be clarified that, in all 6 cases, the diagnosis of *Legionella pneumophila* pneumonia was mainly based on mNGS examination, which may be insufficient. In addition, from these 6 cases, it was not evident to assume that omadacycline was equally effective with other antibiotic therapies in patients with *Legionella pneumonia* infection. However, their liver function was improved after switching to omadacycline. Therefore, omadacycline might be a safe alternative among patients with liver impairment, and its efficacy should be further validated.

## 4 Conclusion

The present study reports the clinical success of 6 patients with *Legionella pneumophila* pneumonia receiving omadacycline after experiencing liver dysfunction. Inspiringly, this case series provides evidence that close monitoring of liver enzymes is required when initiating empiric antibiotics. This study also implies that omadacycline may serve as an optional antibiotic for patients with *Legionella pneumophila* pneumonia after experiencing abnormal liver function. However, more clinical studies are required to validate the efficacy and safety of omadacycline in patients with *Legionella pneumophila* pneumonia.

## Data availability statement

The original contributions presented in the study are included in the article/supplementary material, further inquiries can be directed to the corresponding author.

## Ethics statement

The studies involving humans were approved by ethics committee of Xi’an No. 3 Hospital, The Affiliated Hospital of Northwest University. The studies were conducted in accordance with the local legislation and institutional requirements. The participants provided their written informed consent to participate in this study.

## Author contributions

AZ: Data curation, Formal analysis, Investigation, Methodology, Writing – original draft. QM: Data curation, Formal analysis, Investigation, Methodology, Resources, Writing – original draft. ZL: Conceptualization, Resources, Supervision, Validation, Writing – review & editing.
